# Evaluation of Cervical Lymph Node Metastasis in Papillary Thyroid Carcinoma Using Clinical-Ultrasound Radiomic Machine Learning-Based Model

**DOI:** 10.3390/cancers14215266

**Published:** 2022-10-26

**Authors:** Enock Adjei Agyekum, Yong-Zhen Ren, Xian Wang, Sashana Sashakay Cranston, Yu-Guo Wang, Jun Wang, Debora Akortia, Fei-Ju Xu, Leticia Gomashie, Qing Zhang, Dongmei Zhang, Xiaoqin Qian

**Affiliations:** 1 Department of Ultrasound, Jiangsu University Affiliated People’s Hospital, Zhenjiang 212002, China; 2 School of Medicine, Jiangsu University, Zhenjiang 212002, China; 3 Department of Ultrasound, Nanjing Lishui District Hospital of Traditional Chinese Medicine, Nanjing 211200, China; 4 Department of Biomedical Engineering, School of Communication and Information Engineering, Shanghai University, Shanghai 200444, China; 5 Department of Clinical Microbiology, School of Medicine and Dentistry, Kwame Nkrumah University of Science and Technology, Kumasi 00233, Ghana; 6 Department of Imaging, Klintaps University College, Accra 00233, Ghana

**Keywords:** ultrasound radiomics, papillary thyroid carcinoma, cervical lymph node metastasis, thyroid neoplasms, machine learning

## Abstract

**Simple Summary:**

Accurate preoperative cervical lymph node metastasis (CLNM) prediction in papillary thyroid cancer (PTC) patients is essential for clinical treatment effectiveness, particularly for surgeons assessing the degree of surgical resection and the requirement for cervical lymph node dissection. As a result, a definite diagnosis of CLNM before surgery can assist the surgeon in selecting the best surgical technique and reducing the likelihood of reoperation. This research used several machine learning models based on clinical risk factors in conjunction with radiomics features to preoperatively evaluate CLNM in PTC patients, which can assist clinicians to choose a suitable treatment strategy for patients.

**Abstract:**

We aim to develop a clinical-ultrasound radiomic (USR) model based on USR features and clinical factors for the evaluation of cervical lymph node metastasis (CLNM) in patients with papillary thyroid carcinoma (PTC). This retrospective study used routine clinical and US data from 205 PTC patients. According to the pathology results, the enrolled patients were divided into a non-CLNM group and a CLNM group. All patients were randomly divided into a training cohort (*n* = 143) and a validation cohort (*n* = 62). A total of 1046 USR features of lesion areas were extracted. The features were reduced using Pearson’s Correlation Coefficient (PCC) and Recursive Feature Elimination (RFE) with stratified 15-fold cross-validation. Several machine learning classifiers were employed to build a Clinical model based on clinical variables, a USR model based solely on extracted USR features, and a Clinical-USR model based on the combination of clinical variables and USR features. The Clinical-USR model could discriminate between PTC patients with CLNM and PTC patients without CLNM in the training (AUC, 0.78) and validation cohorts (AUC, 0.71). When compared to the Clinical model, the USR model had higher AUCs in the validation (0.74 vs. 0.63) cohorts. The Clinical-USR model demonstrated higher AUC values in the validation cohort (0.71 vs. 0.63) compared to the Clinical model. The newly developed Clinical-USR model is feasible for predicting CLNM in patients with PTC.

## 1. Introduction

Thyroid cancer is the most common endocrine neoplasm and the twelfth most common type of cancer [1]. Papillary thyroid carcinoma (PTC) is the most common subtype, accounting for approximately 80% of confirmed cases [2,3,4,5,6] and at least 85% of all well-differentiated follicular thyroid cancers. Studies have revealed that lymph node metastasis is linked to disease recurrence and that it occurs in between 30% and 80% of PTC patients [7,8,9]. Cervical lymph node metastasis (CLNM) must be identified preoperatively and postoperatively in patients with thyroid cancer since its detection is crucial for limiting tumor recurrence and survival [10,11,12,13]. Ultrasound (US) is a safe and non-invasive imaging tool that can be used to count thyroid nodules as a first step [14]. A real-time dynamic exploration of the internal structure of the lesion and neighboring sections of the US has its own distinct advantages when compared to other examination methods. At the same time, high-frequency US is becoming a preferred method for detecting and diagnosing PTC patients’ CLNM, as previous research has shown that preoperative US can aid in the diagnosis of PTC CLNM; however, some diagnostic limitations in US evaluation are unavoidable [15,16].

The accuracy of US diagnosis is heavily dependent on different operators’ experience and subjective judgment, different interpretations of the same image, and different machine parameters. Moreover, it is worth noting that the human body contains approximately 800 lymph nodes, 300 of which are located in the head and neck. Because the lymphatic vessels and lymph nodes in the head and neck are so abundant, when PTC metastasizes along the lymphatic vessels to the cervical lymph nodes, there are often multiple metastases. It is difficult to detect all metastatic lymph nodes through imaging [15]. Furthermore, patients with suspicious lymph nodes must undergo fine-needle biopsy and preventive lymph node dissection to find lymph node metastasis. However, both processes are invasive and unnecessary for most patients without lymph node metastasis, and surgical complications such as hypoparathyroidism and recurrent laryngeal nerve injury will severely impact patients’ quality of life [17]. As a result, it is critical to develop a more objective and accurate method for predicting CLNM in PTC patients prior to surgery in clinical practice. US radiomics (USR) is a new tool that can extract hundreds of quantitative features from medical images and combine them into a radiomic signature, an image-based biomarker that can be used to diagnose diseases [18,19]. Machine learning based on radiomics is quickly gaining ground in the medical field [20]. It aims to detect patterns in imaging data and provide decision support by linking these patterns to treatment outcomes, allowing for greater precision in diagnosis and prognosis [21,22].

Clinical risk factors and the USR combined machine learning model, which incorporates clinical and radiomic features, can help provide complementary information for image features and collaborate to improve model performance by combining clinical and US image features. As a result, the combination of clinical risk factors and USR may be able to extract more valuable information from PTC US images, providing better prediction. The purpose of this research is to evaluate the diagnostic performance of a Clinical-USR machine learning model on conventional PTC US images in predicting CLNM in PTC patients.

## 2. Materials and Methods

**Figure 1:**
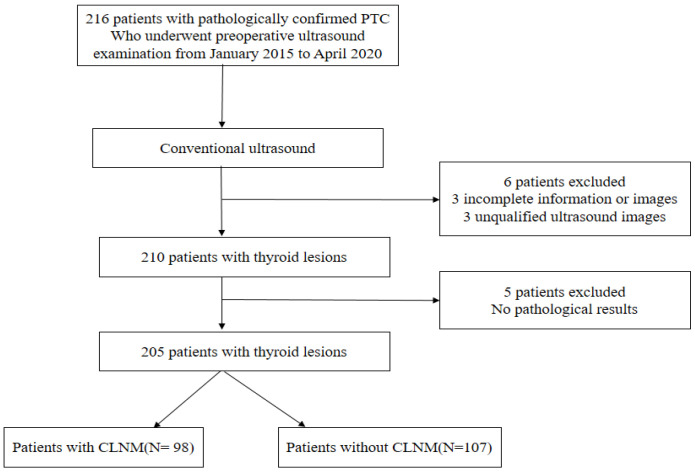
Schematic diagram of the patient selection. PTC, papillary thyroid carcinoma.

The local ethics committee at Jiangsu University’s Affiliated People’s Hospital approved the retrospective study, and informed consent was waived. In total, 205 patients from our hospital were selected retrospectively between January 2015 and April 2020. The enrollment process is depicted in Figure 1.

All patients had routine 2-dimensional US. The training cohort (*n* = 143) and validation cohort (*n* = 62) were divided in a 7:3 ratio. The included cases were divided into CLNM and CLNM-free groups based on pathological results. The inclusion criteria were (1) nodules that had clear surgery- and puncture biopsy pathology-confirmed PTC; (2) routine US, with complete images, clear quality; and (3) did not receive chemoradiotherapy or other cancer treatment prior to surgery. Exclusion criteria were (1) pathology results that could not be identified as PTC nodules; (2) unclear images with incomplete nodules; (3) pregnant and lactating women; and (4) patients with a severe allergic history or severe cardiopulmonary disease.

### 2.1. Ultrasound Examination

All patients had a routine US examination prior to surgery, which was performed by well-trained technicians using a Philips Q5, Philips iU22 (both Healthcare, Eindhoven, The Netherlands) or a GE LOGIQ s8, LOGIQ E20, LOGIQ E9 (GE Medical Systems, American General, Boston, MA, USA) US system with a 5–12 MHz linear array transducer. The patient was positioned supine with the pillow removed in order to lower and slightly recline the head. This exposed as much of the neck as possible in order to perform a US examination of the thyroid and cervical area using longitudinal, horizontal continuous scanning and carefully scanning the lymph nodes in all areas of the neck.

This allowed for the measurement of thyroid tumor size or mass (maximum long axis of the nodule), tumor location (left lobe, right lobe, or isthmus), tumor position (upper, middle, or lower pole), aspect ratio (≤1 or >1), internal echo pattern (uniform or nonuniform), tumor border (clear, less clear), shape (regular or irregular), US ETE diagnosis (without ETE or with ETE)**,** tumor peripheral blood flow (without or abundant), and tumor internal vascularization (without or abundant).

### 2.2. Region of Interests (ROIs) Segmentation

ROIs were manually drawn on US images by one radiologist with 15 years of experience in thyroid disease diagnosis using the software ITK-SNAP (version 3.8.0, http://www.itksnap.org. Accessed 10 August 2021) to indicate focal areas within the thyroid gland. The ROI was applied to the tumor’s solid component, avoiding necrotic, hemorrhagic, and cystic areas. Thirty patients were chosen at random to assess the consistency of the ROI placements, and a second physician with 8 years of experience in thyroid US diagnosis independently placed ROIs on the relevant structures.

### 2.3. Radiomic Features Extraction

In total, 1046 USR features were extracted from these ROIs on US images using PyRadiomics (Version 2.2.0, https://github.com/Radiomics/pyradiomics. Accessed 10 August 2021). These features included first-order features, shape features, grey-level run length matrix (GLRLM) features, grey-level size zone matrix (GLSZM) features, grey-level dependence matrix (GLDM) features, neighborhood grey-tone dependency matrix (NGTDM) features, grey-level co-occurrence matrix (GLCM) features, and features derived from wavelet filter images containing first-order GLCM, GLRLM, GLSZM, GLDM, and NGTDM features.

### 2.4. Feature Selection and Construction of USR Model

To reduce bias and over-fitting in the study, the extracted features were normalized using a standard scalar. Because of the high dimensionality of the feature space, the number of features must be reduced to avoid the interference of a large number of redundant features in the data analysis, which has an impact on model construction and raises computational costs. The dataset was partitioned into training and validation cohorts.

We used Pearson’s Correlation Coefficient (PCC) to reduce the row spatial dimension of the feature matrix so that each characteristic is relatively independent of the training data. Any two features with a PCC greater than 0.85 were considered redundant. Finally, in the training cohort, representative features were chosen using Recursive Feature Elimination (RFE) with 15-fold cross-validation. The Support Vector Machine with the linear kernel (SVM-L) [23], Support Vector Machine with radial basis function kernel (SVM-RBF) [23], LogisticRegressionCV (LRCV) [23], and Linear Discriminant Analysis (LDA) [23] classifiers were used to build the prediction models using the RFE’s key features. In the validation procedure, the same feature sets were selected, and they were fed into the model.

**Figure 2:**
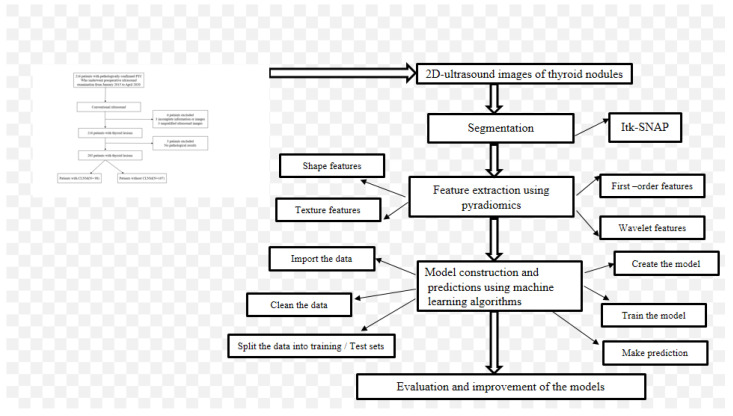
Schematic diagram of the radiomic workflow in building the machine learning models.

The model’s performance on the training and validation datasets was assessed using standard clinical statistics such as the area under the curve (AUC), sensitivity, specificity, negative predictive value (NPV), positive predictive value (PPV), and accuracy. The set of models that performed the best on the validation dataset was identified and evaluated by comparing diagnostic performance, and the best classifier was chosen. Figure 2 depicts the radiomic and machine learning workflow.

### 2.5. Construction of Clinical Model

In this study, basic clinical characteristics were analyzed, and then statistically significant characteristics with *p* < 0.05 were chosen to construct a Clinical Model, using SVM-L, SVM-RBF, LRCV, and LDA classifiers. The AUC and other relevant statistics were calculated to evaluate the diagnostic efficiency.

### 2.6. Development of the Clinical-USR Model

Statistically significant clinical factors were combined with extracted USR features to form a new feature set. A Clinical-USR model incorporating USR features and clinical characteristics was built using SVM-L, SVM-RBF, LRCV, and LDA classifiers, and the diagnostic efficacy of the combined prediction model was evaluated. The precision-recall curve shows the trade-off between precision and recall for different thresholds. High precision relates to a low false positive rate, and high recall relates to a low false negative rate.

High scores for both show that the classifier returns accurate results (high precision), as well as returning the majority of all positive results (high recall). Average precision (AP) summarizes such a plot as the weighted mean of precisions achieved at each threshold, with the increase in recall from the previous threshold used as the weight [23]. The precision–recall analysis of the model was assessed using the precision–recall curve. We also investigated the rate of false positives and false negatives, using the confusion matrix.

### 2.7. Statistical Analysis

Statistical analyses were carried out using Python (version 3.7 https://www.python.org/. Accessed 8 July 2021) and IBM SPSS Statistics for Windows version 26.0. (Armonk, NY, USA). To compare differences in categorical characteristics, Pearson’s chi-square or Fisher’s exact test were used. For continuous factors with normal distribution, the independent sample *t*-test was used, whereas, for continuous factors without normal distribution, the Mann–Whitney U test was used. A two-sided *p* < 0.05 indicated statistically significant differences.

PyRadiomics (version 2.2.0, https://github.com/Radiomics/pyradiomics. Accessed 10 August 2021) and scikit-learn version 1.2 [23] were used to extract USR features and build the prediction models. The AUC, sensitivity, specificity, accuracy, NPV, and PPV of each prediction model were calculated. The scikit-learn version 1.2 [23] was used to draw the precision–recall curve.

## 3. Results

### 3.1. Clinical Characteristics

A total of 205 patients with PTC were enrolled from ages 18–78 years, with an average age of 47.22 ± 11.30 years, and a male-to-female ratio of 1:3.56. CLNM was excluded in 107 patients and confirmed in 98. Using stratified sampling, all patients were randomly assigned to a training group (*n* = 143) and a validation group (*n* = 62). Table 1 displays the clinical data for the training and validation groups. Table 2 displays the clinical data for the CLNM and non-CLNM groups. Age, US CLNM diagnosis, mass, capsular invasion, shape, US ETE diagnosis, internal echo, aspect ratio, and tumor internal vascularization (all *p* < 0.05) did differ significantly between the two groups.

### 3.2. Clinical Model Construction

**Figure 3:**
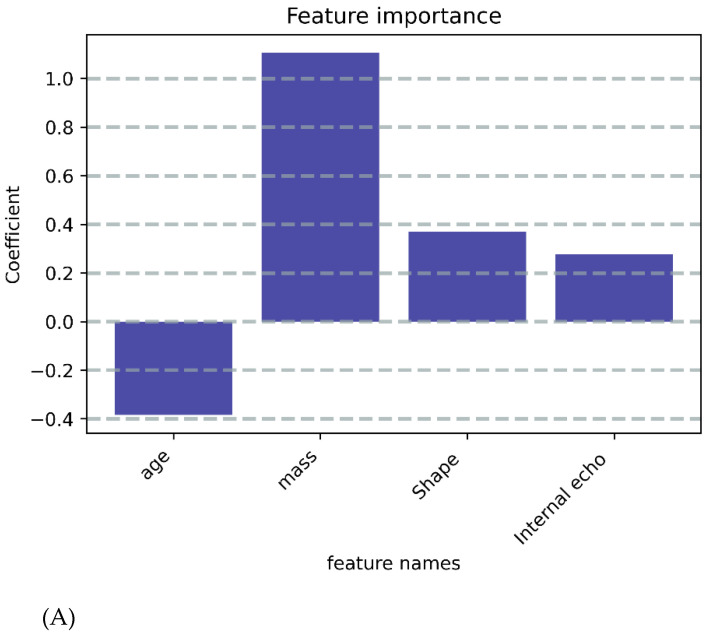

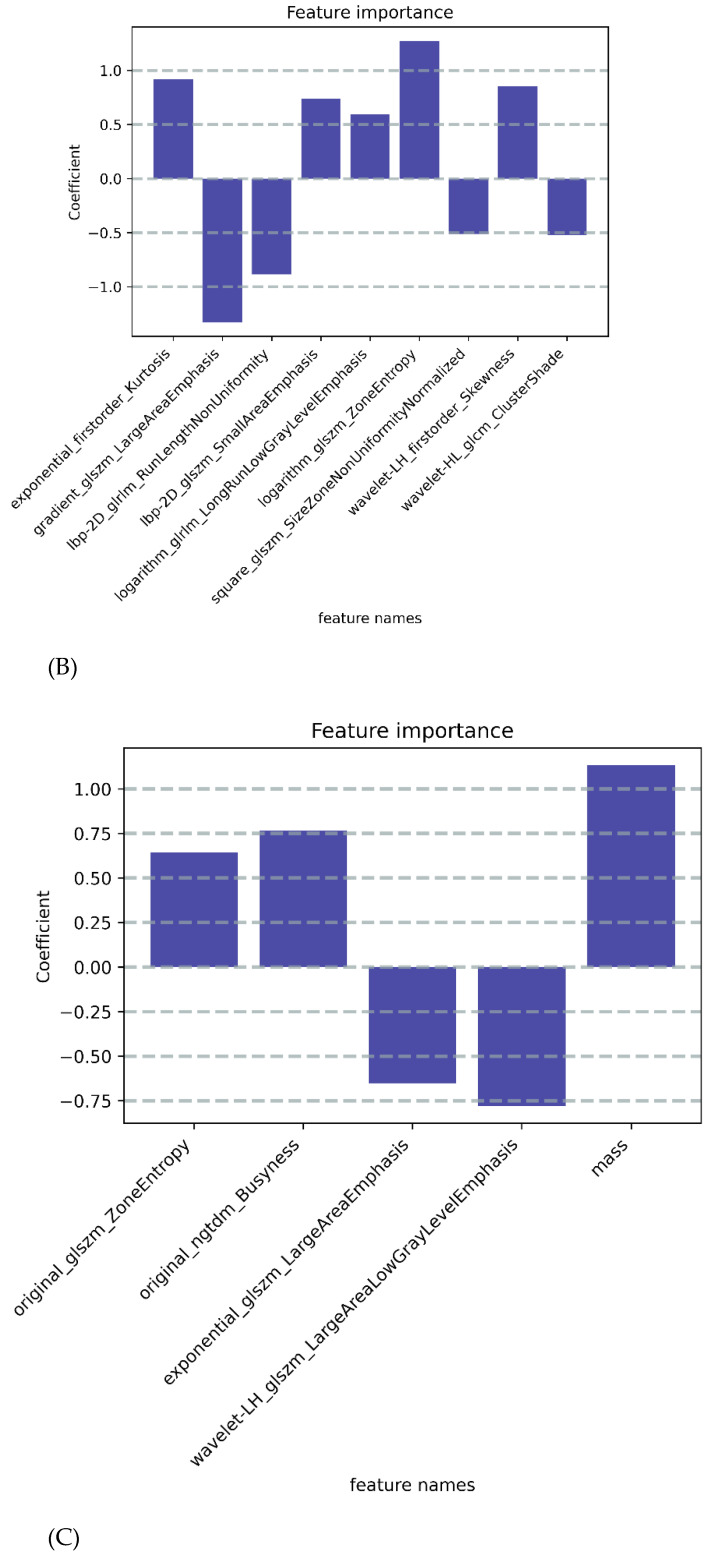
**Selected features after RFE.** (**A**) In the Clinical model, features were reduced to four features in the training cohort. (**B**) In the USR model, features were reduced to nine features in the training cohort. (**C**) In the Clinical-USR model, features were reduced to five features in the training cohort.

**Figure 4:**
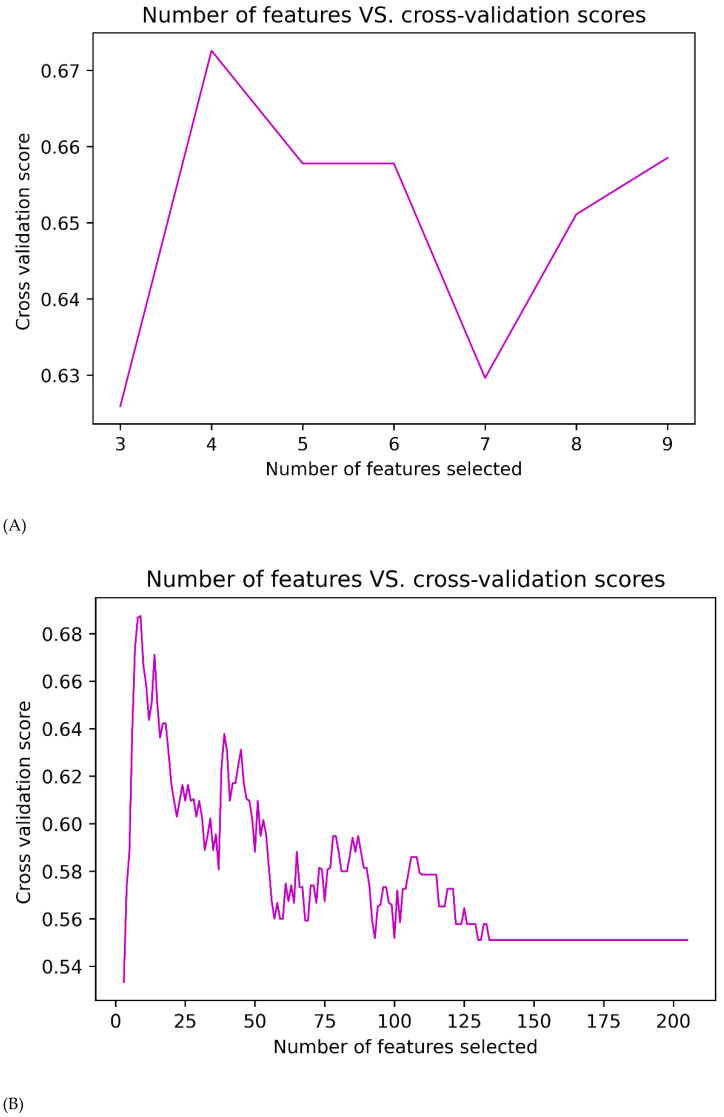

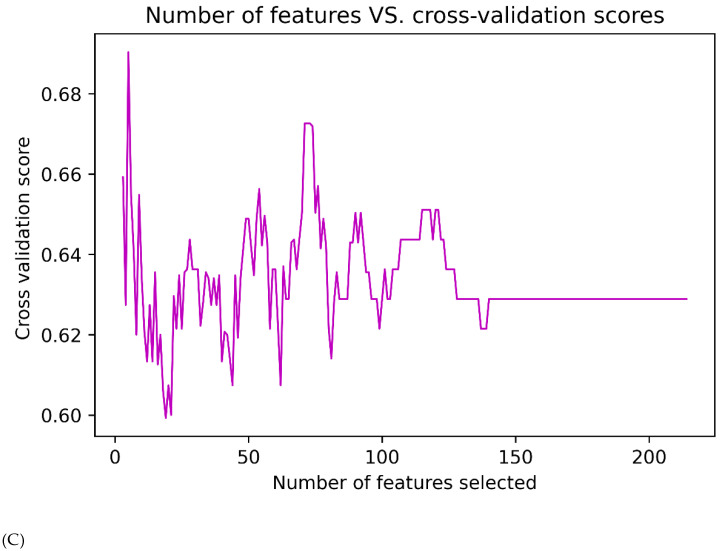
Recursive feature elimination (RFE) with 15-fold cross-validation; number of features selected vs. cross-validation score. (**A**) Clinical model. (**B**) USR model. (**C**) Clinical-USR model.

A clinical model was built using nine variables that were statistically significant. RFECV was also used to select the best variables, and the final four key variables that were chosen were age, mass, shape, and internal echo (Figure 3A–C and Figure 4A–C).

**Figure 5:**
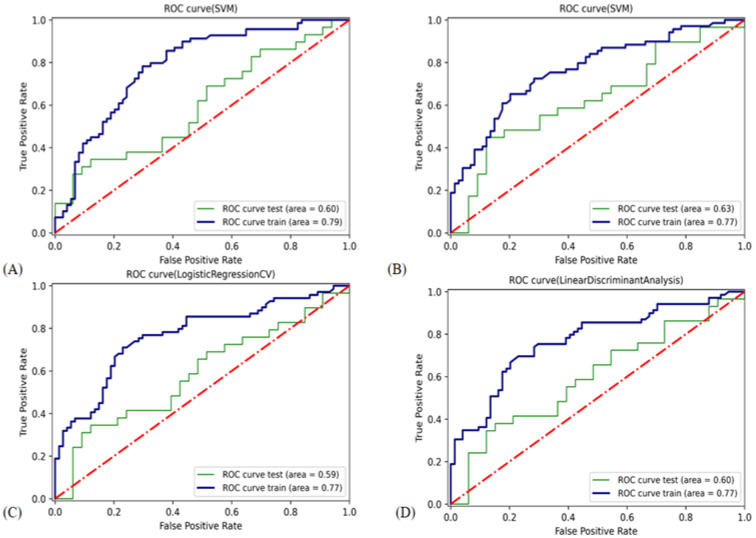
ROC curve of the classifiers used in building the Clinical model. ROC, receiver operating characteristic. (**A**) Support vector classifier with radial basis function kernel. (**B**) Support vector classifier with linear kernel. (**C**) Logistic regression classifier. (**D**) Linear discriminant analysis.

The SVM-L classifier had the highest AUC (0.63) in the validation cohort compared with the other classifiers, so it was selected as the Clinical model (Table 3 and Figure 5).

**Figure 6:**
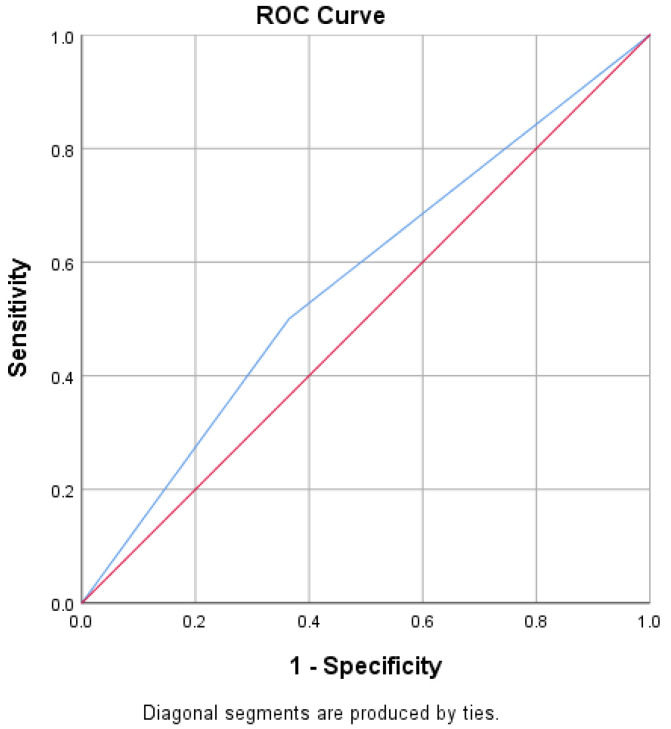
ROC curve of the US reported status of CLNM by an experienced sonographer. ROC, receiver operating characteristic.

Based on US findings evaluated by an experienced sonographer, the US-reported status of CLNM by an experienced sonographer in directly detecting suspicious malignant cervical lymph node had an AUC of 0.568 (Figure 6), a sensitivity of 0.500, specificity of 0.636, NPV of 0.581, and PPV of 0.557.

### 3.3. Construction of USR Model

A total of 1046 USR features were extracted from each 2-D US image. Any two features with a PCC > 0.85 were considered redundant. Nine features were selected in the training cohort using RFE and stratified 15-fold cross-validation (Figure 3B and Figure 4B).

**Figure 7:**
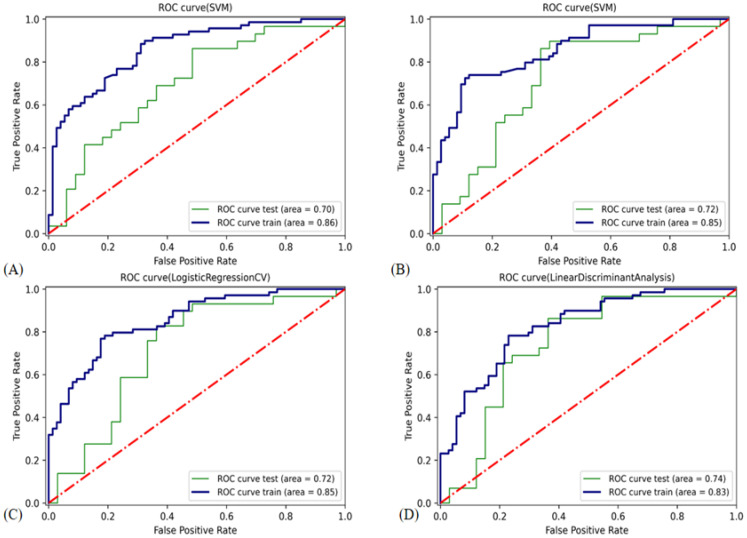
ROC curve of the classifiers used in building the USR model. ROC, receiver operating characteristic. (**A**) Support vector classifier with radial basis function kernel. (**B**) Support vector classifier with linear kernel. (**C**) Logistic regression classifier. (**D**) Linear discriminant analysis.

The features chosen were exponential_firstorder_Kurtosis, gradient_glszm_LargeAreaEmphasis, lbp_2D_glrlm_RunLengthNonUniformity, lbp_2 D_glszm_SmallAreaEmphasis, logarithm_glrlm_LongRunLowGrayLevelEmphasis, logarithm_glszm_ZoneEntropy, square_glszm_SizeZoneNonUniformityNormalized, wavelet_LH_firstorder_Skewness, and wavelet_HL_glcm_ClusterShade. In the validation cohort, the LDA classifier had the highest AUC (0.74) value compared with the other classifiers. The LDA was chosen for the USR model. Figure 7 shows the ROC curves of the USR model using various classifiers. Table 3 displays detailed information about the classifiers’ prediction performance. LDA decision function is given as follows:

Decision value = 0.919 × exponential_firstorder_Kurtosis − 1.327 × gradient_glszm_LargeAreaEmphasis − 0.886 × lbp_2D_glrlm_RunLengthNonUniformity + 0.736 × lbp_2D_glszm_SmallAreaEmphasis + 0.596 × logarithm_glrlm_LongRunLowGrayLevelEmphasis + 1.273 × logarithm_glszm_ZoneEntropy − 0.512 × square_glszm_SizeZoneNonUniformityNormalized + 0.856 × wavelet_LH_firstorder_Skewness − 0.520 × wavelet_HL_glcm_ClusterShade.

### 3.4. Construction of Clinical-USR Model

We created a new feature set by combining the extracted USR features and nine clinical parameters.

Through PCC and RFE (Figure 4C), five key features, namely original_glszm_ZoneEntropy, original_ngtdm_Busyness, exponential_glszm_LargeAreaEmphasis, wavelet-LH_glszm_LargeAreaLowGrayLevelEmphasis, and mass (Figure 3C, Table 4) were selected to construct the Clinical-USR model.

**Figure 8:**
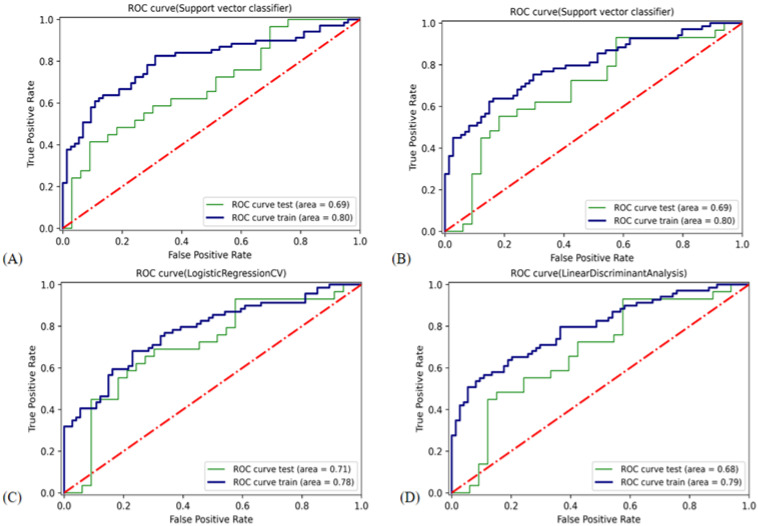
ROC curve of the classifiers used in building the Clinical-USR model. ROC, receiver operating characteristic. (**A**) Support vector classifier with radial basis function kernel. (**B**) Support vector classifier with linear kernel. (**C**) Logistic regression classifier. (**D**) Linear discriminant analysis.

In the validation cohort, the LRCV classifier had the highest AUC (0.71) and ACC (0.68) values compared with the other three classifiers. The LRCV was chosen for the Clinical- USR model. Figure 8 shows the ROC curves of the Clinical- USR model using various classifiers.

Detailed information about the prediction performance of the classifiers is shown in Table 3.

LRCV decision function of the Clinical-USR model is given as follows:

Decision value = 0.642 × original_glszm_ZoneEntropy + 0.766 × original_ngtdm_Busyness − 0.652 × exponential_glszm_LargeAreaEmphasis − 0.782 × waveletLH_glszm_LargeAreaLowGrayLevelEmphasis + 1.132 × mass.

**Figure 9:**
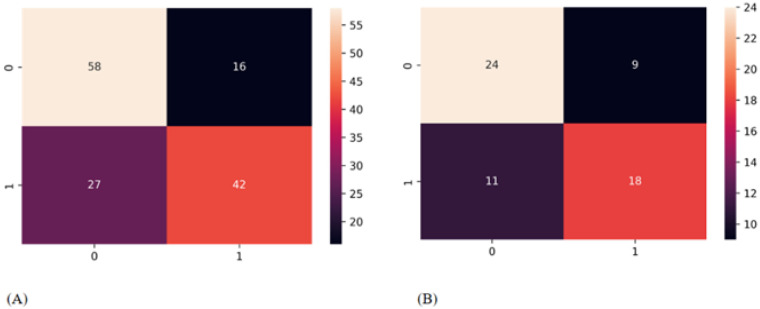
Confusion matrix. The 2 × 2 contingency table reports the number of true positives, false positives, false negatives, and true negatives: Training cohort (**A**) and validation cohort (**B**).

On the training cohort containing 143 patients, the classifier correctly classified 42 (true positive) out of 69 without CLNM, and correctly classified 58 (true negative) out of 74 patients having CLNM (Figure 9A,B).

**Figure 10:**
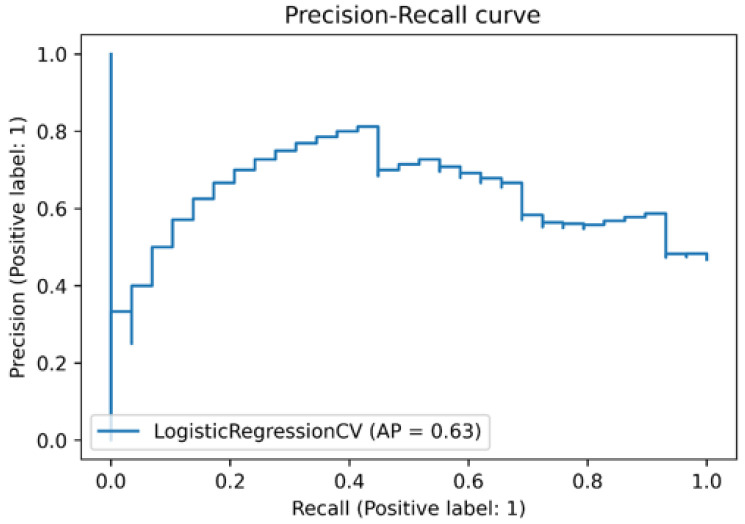
Precision–recall curve of the Clinical-USR model.

In the validation cohort containing 62 patients, the Clinical-USR model correctly classified 18 (true positive) out of 29 patients with no CLNM, and correctly classified 24 (true negative) out of 33 patients having CLNM (Figure 9B)**.** The precision–recall curve of the Clinical-USR model is shown in Figure 10.

Further multivariate analysis of the features used in building the Clinical-USR model indicated that original glszm ZoneEntropy, original ngtdm Busyness, WavletLH glszm LargeAreaLow GrayLevelEmphasis, and mass are independent predictors of CLNM in PTC (Table 5).

## 4. Discussion

Early diagnosis of tumors with lymph node metastatic potential is crucial for preventing the fast progression of PTC. It also serves as a key marker for monitoring PTC progression and a predictor of a poor prognosis. Surgery is presently the first-line therapy for PTC patients who have received a clinical diagnosis. Individuals with a suspected risk of CLNM must have cervical preventative lymph node dissection; nevertheless, it is questionable what PTC patients can gain from prophylactic lymph node dissection. According to studies, early detection of lymph node metastases results in fewer recurrences and a better prognosis in PTC patients, who have a higher chance of surviving cancer [10,12]. People with PTC who have CLNM have higher rates of death and recurrence than patients without CLNM [24，^25^]. Furthermore, surgery might potentially impair postoperative parathyroid function and increase the chance of laryngeal recurrent nerve damage. It is also more strenuous on the surgeon and demands a greater degree of skill [26,27]. Therefore, a definitive diagnosis of CLNM before surgery can help the surgeon choose the optimal surgical strategy and reduce the chance of reoperation.

For clinical treatment to be effective, accurate preoperative CLNM prediction in PTC patients is crucial, especially for the surgeons to assess the extent of surgical resection and the need for cervical lymph node dissection [28,29]. In many patients, CLNM may not reveal any aberrant findings in preoperative US examination [30] due to US's unreliability in visualizing deep anatomic structures or structures that are acoustically shadowed by air and bone [31]. Additionally, because US examination is an empirical diagnostic that is greatly impacted by the operator's experience, it is subject to interobserver variability when determining CLNM [32]. We employed the Clinical-USR approach to identify CLNM before surgery in our study. The developed Clinical-USR model is a simple-to-use diagnostic and prognostic tool that could help patients without CLNM avoid unnecessary surgery. The AUC values revealed that the Clinical-USR model could discriminate between PTC patients with CLNM and PTC patients without CLNM in the training (AUC: 0.78) and validation (AUC: 0.71) cohorts. When compared to the Clinical model, the USR model had higher AUCs in both the training (0.83 vs. 0.77) and validation (0.74 vs. 0.63) cohorts. In both the training (AUC: 0.78 vs. 0.77) and validation (AUC: 0.71 vs. 0.63) cohorts, the Clinical-USR model showed higher AUC values than the Clinical model.

Original glszm ZoneEntropy, original ngtdm Busyness, exponential glszm LargeAreaEmphasis, and wavelet LH glszm LargeAreaLowGrayLevelEmphasis are the selected USR features that were used in constructing the Clinical-USR model. In an image, GLSZM measures gray level zones. The number of related voxels with the same gray-level intensity is defined as a gray-level zone. If the distance between two voxels is 1 according to the infinity norm, they are considered linked (26 connected regions in 3D, 8 connected regions in 2D) [33]. The surrounding gray-tone difference matrix is used to calculate the difference between a gray value and its close neighbors’ average gray values [34]. The distribution of zone widths and gray levels is measured for uncertainty/randomness using original glszm ZoneEntropy. More heterogeneity in the texture patterns is indicated by a higher value. Exponential glszm LargeAreaEmphasis is a measurement of how big area size zones are distributed, with a higher value indicating larger size zones and coarser textures.

Wavelet-LH glszm LargeAreaLowGrayLevelEmphasis glszm measures the fraction of the image’s combined distribution of bigger size zones with lower gray-level values. Original ngtdm Busyness is a measure of the difference between a pixel and its neighbor. A high busyness rating suggests a ‘busy’ image, with fast changes in intensity between pixels and their surroundings.

This suggests that the newly constructed Clinical-USR model contains additional data that are significantly associated with PTC with CLNM but not a standard risk factor. PTC density and increased nonuniformity, for example, are features that are difficult to measure with the naked eye. However, in PTC, these characteristics are linked to tissue heterogeneity. For CLNM evaluation, the Clinical-USR model considers PTC heterogeneity, a measurable trait associated with the degree of malignancy in PTC. As a result, the quantitative Clinical-USR approach not only overcomes the subjectivity of standard US imaging diagnosis, but also makes use of a great deal of data that the naked eye cannot see.

According to postoperative pathology results, the US-reported status of CLNM by an experienced sonographer in directly detecting suspicious malignant cervical lymph node had an AUC of 0.568, a sensitivity of 0.500, specificity of 0.636, NPV of 0.581, and PPV of 0.557. There are approximately 200 lymph nodes distributed in the neck. Because the lymphatic vessels and lymph nodes in the neck are so numerous, when PTC metastasizes to the cervical lymph nodes, multiple metastases are common, and it is difficult to detect all metastatic lymph nodes with US. In this study, the clinical-USR model outperformed an experienced sonographer’s US-reported CLNM status.

Li et al. [35] developed a deep learning-based computer-aided model for the diagnosis of CLNM in patients with PTC, and then tested the model’s accuracy with a validation set. In the validation cohort, the AUC of their model for the diagnosis of CLNM was higher than that of the current study (0.79 vs. 0.71). The reason for this could be the deep learning approach they used in their research. In our research, we used machine learning. Most of the applicable features in traditional machine learning approaches must be identified by a domain expert in order to minimize data complexity and make patterns more obvious for learning algorithms to work. Deep learning algorithms have the biggest benefit in that they aim to learn high-level features from data incrementally. This reduces the need for domain expertise and the extraction of hardcore features.

In their study, Tian et al. [36] developed a CLNM prediction model based on clinical risk variables. The AUC of their model for the diagnosis of CLNM in the validation cohort was slightly lower than that of the current study (0.70 vs. 71). Zou et al. [37] used machine learning models based on US data to determine the probability of CLNM. The AUC of their model for the diagnosis of CLNM was slightly higher in the validation cohort compared with the current study (0.73 vs. 0.71). The slightly higher AUCs in the preceding studies could be attributed to the larger number of patients included in their studies. Large datasets improve predictive model performance in machine learning. Many studies, including the abovementioned studies, have focused on predicting CLNM based on either US clinical risk factors or USR features, but not their combination. We developed a joint predictive model incorporating clinical risk factors and USR features, using four different machine learning algorithms in our study. In this study, we also built three different models: The Clinical model, the USR model, and the Clinical-USR model, and we employed and compared the performances of several machine learning classifiers in the construction of each model, and the best-performing classifier was chosen to represent the models. One statistic for evaluating the excellence of a model’s output is precision–recall. While recall assesses the number of really relevant results returned, precision evaluates the relevance of the results. In the stairstep portion of Figure 6, recall and precision are inversely correlated; near the margins of these steps, a tiny adjustment in the threshold significantly affects precision while only slightly increasing recall. From the precision–recall analysis of the Clinical-USR model, the AP of the model was 0.63, which is satisfactory.

## 5. Conclusions

Because this was a retrospective study, there may have been a case selection bias that influenced the study results. Furthermore, we established and validated our Clinical-USR model for distinguishing CLNM in a single hospital and employed only greyscale US images in our Clinical-USR model. We do, however, intend to integrate multimodal USR characteristics in future studies. In summary, for the prediction of CLNM, a Clinical-USR model based on clinical factors and USR features was developed. The newly developed Clinical-USR model is non-invasive and feasible for predicting CLNM in PTC patients.

## Figures and Tables

**Table 1:** Patient characteristics of the training and validation cohorts.

Characteristic	Training Cohort (*n* = 143)	Validation Cohort (*n* = 62)	*p*
**Age**, mean ± SD, years	47.29 ± 11.31	47.06 ± 11.36	0.981
**Sex, *n***			
Female	114	46	
Male	29	16	0.242
Ultrasound Characteristic			
**Tumor size** (mass)	9.22 ± 6.85	9.61 ± 6.70	0.662
**Tumor location**			
Left lobe	60	18	
Right lobe	48	29	
Isthmus	35	15	0.142
**Tumor position**			
Upper pole	73	25	
Middle pole	5	2	
Inferior pole	65	35	0.346
**Internal echo pattern**			0.312
Uniform	29	10	
Nonuniform	114	52	
**Tumor border**			0.015 *
Clear	48	11	
unclear	95	51	
**Tumor internal vascularization**			
Without	75	38	0.155
Abundant	68	24	
**Tumor Peripheral blood flow**			
Without	80	31	0.263
Abundant	63	31	
**Ultrasound ETE diagnosis**			0.495
Without ETE	133	57	
With ETE	10	5	
**Aspect ratio**			0.434
≤1	91	38	
>1	52	24	
**Shape**			0.380
Regular	106	44	
Irregular	37	18	
**Ultrasound CLNM diagnosis**			0.367
Without CLNM	80	37	
With CLNM	63	25	
Postoperative diagnosis			
**capsular invasion**			0.432
Negative	75	31	
Positive	68	31	

ETE, extrathyroidal extension; CLNM, cervical lymph node metastasis; SD, standard deviation. *: statistically significant.

**Table 2:** Patient characteristics of the PTC with CLNM and PTC without CLNM groups.

Characteristic	CLNM (−) (*n* = 107)	CLNM (+) (*n* = 98)	*p*
**Age**, mean ± SD, years	48.97 ± 10.81	45.31 ± 11.56	0.035 *
**Sex, *n***			
Female	80	80	
Male	27	18	0.154
Ultrasound Characteristic			
**Tumor size** (mass)	7.30 ± 5.01	11.57 ± 7.74	0.00 *
**Tumor location**			
Left lobe	44	34	
Right lobe	40	37	
Isthmus	23	27	0.515
**Tumor position**			
Upper pole	53	45	
Middle pole	4	3	
Inferior pole	50	50	0.818
**Internal echo pattern**			0.033 *
Uniform	26	13	
Nonuniform	81	85	
**Tumor border**			0.463
Clear	30	29	
unclear	77	69	
**Tumor internal vascularization**			0.001 *
Without	71	42	
Abundant	36	56	
**Tumor Peripheral blood flow**			0.236
Without	61	50	
Abundant	46	48	
**Ultrasound ETE diagnosis**			0.009 *
Without ETE	104	86	
With ETE	3	12	
**Aspect ratio**			0.000 *
≤1	55	74	
>1	52	24	
**Shape**			0.025 *
Regular	85	65	
Irregular	22	33	
**Ultrasound CLNM diagnosis**			0.035 *
Without CLNM	68	49	
With CLNM	39	49	
Postoperative diagnosis			
**Capsular Invasion**			0.022 *
Negative	63	43	
Positive	44	55	

ETE, extrathyroidal extension; CLNM, cervical lymph node metastasis; SD, standard deviation. *: statistically significant.

**Table 3:** Predictive performance of the machine learning models for the training and validation cohorts.

Training Cohort						Validation Cohort						
	ACC	AUC	SEN	SPEC	PPV	NPV	ACC	AUC	SEN	SPEC	PPV	NPV
**Clinical model**												
LDA	0.71	0.77	0.58	0.82	0.75	0.68	0.56	0.60	0.55	0.58	0.53	0.59
LRCV	0.71	0.77	0.61	0.81	0.75	0.69	0.55	0.59	0.55	0.55	0.52	0.58
SVM-L	0.72	0.77	0.61	0.82	0.76	0.69	0.61	0.63	0.59	0.64	0.59	0.64
SVM-RBF	0.73	0.79	0.78	0.69	0.70	0.77	0.56	0.60	0.69	0.45	0.52	0.63
**USR model**												
LDA	0.76	0.83	0.75	0.77	0.75	0.77	0.66	0.74	0.52	0.79	0.68	0.65
LRCV	0.79	0.85	0.78	0.80	0.78	0.80	0.61	0.72	0.49	0.76	0.62	0.61
SVM-L	0.73	0.85	0.77	0.70	0.71	0.76	0.66	0.72	0.52	0.79	0.68	0.65
SVM-RBF	0.77	0.86	0.77	0.77	0.76	0.78	0.63	0.70	0.78	0.76	0.64	0.63
**Clinical-USR model**												
LDA	0.71	0.79	0.65	0.76	0.71	0.70	0.61	0.68	0.55	0.66	0.60	0.63
LRCV	0.70	0.78	0.61	0.78	0.72	0.68	0.68	0.71	0.62	0.73	0.67	0.69
SVM-L	0.71	0.80	0.65	0.76	0.71	0.70	0.65	0.69	0.59	0.70	0.63	0.66
SVM-RBF	0.73	0.80	0.64	0.82	0.77	0.71	0.63	0.69	0.55	0.70	0.62	0.63

AUC, area under the curve; ACC, accuracy; SEN, sensitivity; SPEC, specificity; NPV, negative predictive value; PPV, positive predictive value; USR, ultrasound radiomic; SVM-L, support vector machine with linear kernel; SVM-RBF, support vector machine with radial basis function kernel; LDA, linear discriminant analysis; LRCV, logistic Regression CV.

**Table 4:** Selected features and their coefficients during the recursive feature elimination procedure.

	Feature Name	Coefficient
original glszm ZoneEntropy	GLSZM	0.642
original ngtdm Busyness	NGTM	0.766
exponential glszm LargeAreaEmphasis	GLSZM	−0.652
waveletLH glszm LargeAreaLowGrayLevelEmphasis	GLSZM	−0.782
Tumor mass	N/A	1.132

N/A: Not applicable.

**Table 5:** Results of multivariate regression analysis of selected features in diagnosis of CLNM in PTC patients.

Feature Name	B	SE	Wald	df	*p*	Exp (B)
Original glszm ZoneEntropy	0.696	0.237	8.627	1	0.003	2.006
Original ngtdm Busyness	1.248	0.546	5.215	1	0.022	3.482
exponential glszm LargeAreaEmphasis	−2.294	2.013	1.298	1	0.254	0.101
WaveletLH glszm LargeAreaLow GrayLevelEmphasis	−896	0.459	3.812	1	0.051	0.408
tumour size(mass)	1.264	0.300	17.726	1	0.000	3.541

## Data Availability

The original contributions presented in the study are included in the article. Further inquiries can be directed to the corresponding authors.
